# Mitigation of UV-B Radiation Stress in Tobacco Pollen by Expression of the Tardigrade Damage Suppressor Protein (Dsup)

**DOI:** 10.3390/cells13100840

**Published:** 2024-05-15

**Authors:** Cecilia Del Casino, Veronica Conti, Silvia Licata, Giampiero Cai, Anna Cantore, Claudia Ricci, Silvia Cantara

**Affiliations:** 1 Dipartimento di Scienze della Vita, University of Siena, via Mattioli 4, 53100 Siena, Italy; delcasino@unisi.it (C.D.C.); silvialicata2@gmail.com (S.L.); 2 Dipartimento di Scienze Biologiche, Geologiche e Ambientali, Università di Bologna, via Irnerio 42, 40126 Bologna, Italy; veronica.conti8@unibo.it; 3 Department of Medical, Surgical and Neurological Sciences, University of Siena, Viale Bracci, 53100 Siena, Italy; anna.cantore@unisi.it (A.C.); claudia.ricci@unisi.it (C.R.); cantara@unisi.it (S.C.)

**Keywords:** UV-B stress, tardigrade damage suppressor protein (Dsup), pollen tube length, cell wall, stress tolerance

## Abstract

Pollen, the male gametophyte of seed plants, is extremely sensitive to UV light, which may prevent fertilization. As a result, strategies to improve plant resistance to solar ultraviolet (UV) radiation are required. The tardigrade damage suppressor protein (Dsup) is a putative DNA-binding protein that enables tardigrades to tolerate harsh environmental conditions, including UV radiation, and was therefore considered as a candidate for reducing the effects of UV exposure on pollen. Tobacco pollen was genetically engineered to express Dsup and then exposed to UV-B radiation to determine the effectiveness of the protein in increasing pollen resistance. To establish the preventive role of Dsup against UV-B stress, we carried out extensive investigations into pollen viability, germination rate, pollen tube length, male germ unit position, callose plug development, marker protein content, and antioxidant capacity. The results indicated that UV-B stress has a significant negative impact on both pollen grain and pollen tube growth. However, Dsup expression increased the antioxidant levels and reversed some of the UV-B-induced changes to pollen, restoring the proper distance between the tip and the last callose plug formed, as well as pollen tube length, tubulin, and HSP70 levels. Therefore, the expression of heterologous Dsup in pollen may provide the plant male gametophyte with enhanced responses to UV-B stress and protection against harmful environmental radiation.

## 1. Introduction

In spermatophytes, the pollen grain forms the pollen tube, which transports male gametes through the pistil tissues. The proper and directed growth of the pollen tube is critical to the fertilization process of seed plants. Pollen tube elongation occurs at the tip of the tube, and the cell growth is dependent on a cooperative interaction between internal turgor pressure and the deposition of cell wall components [1]. The secretory vesicles are produced by the Golgi apparatus to transport cell wall components and enzymes to the tip [2]; here, the fusion of vesicles with the plasma membrane results in the deposition of methyl esterified pectins, which are then converted to acid pectins. This is followed by the deposition of a secondary layer of cellulose and callose, which acts as a structural reinforcement. The formation of “callose plugs” is crucial for confining cytoplasmic material to the growing portion of the pollen tube [3]. As in other plant cells, the proper deposition of cell wall polysaccharides is under the control of the microtubule cytoskeleton; however, this is more relevant for callose because microtubules are likely to control the insertion/activity of callose synthase [4]. On the other hand, the involvement of microtubules in cellulose deposition in the pollen tube cell wall is largely unclear. The transport of male gametes in the pollen tube requires both an intact cytoskeleton and the activity of motor proteins. The interaction of microtubules and actin filaments, as well as the precise role of motor proteins in gamete transport, is unknown, but members of specific kinesin subfamilies are most likely involved [5].

Tardigrades, also known as “water bears”, are small invertebrates that first appeared 500 million years ago. They can inhabit a wide range of habitats, including marine, freshwater, and terrestrial ecosystems. To be active, these organisms require at least a thin layer of water. When water is scarce, they can survive by entering cryptobiosis. During this condition, tardigrades suspend their metabolism and can survive prolonged periods of desiccation (anhydrobiosis) or freezing (cryobiosis) [6]. Anhydrobiosis allows for survival even in harsh environments, such as temperatures as high as 150 °C or as low as absolute zero (−273 °C), as well as during exposure to high levels of ionizing radiation and UV rays [7]. *Ramazzottius varieornatus* is one of the most stress-tolerant tardigrade species. The damage suppressor protein (Dsup) is a typical protein in this organism that has been studied extensively because of its likely association with chromatin and its ability to efficiently protect and repair DNA. Dsup is present in almost all tardigrade cells and is associated with nuclear DNA. When exposed to X-rays, cells expressing Dsup show greater protection against DNA damage and higher cell viability than cells that do not express Dsup [8]. Subsequent research has confirmed the protective role of Dsup when expressed in other cell types, including plants [9,10,11,12].

The UV radiation emitted by the sun is divided into three types based on wavelength: UV-A (400–315 nm), UV-B (315–280 nm), and UV-C (280–100 nm). While UV-C radiation is completely absorbed by the atmosphere, UV-B and UV-A radiation reach the earth’s surface and affect plant organisms in ways that depend on the wavelength and duration of exposure [13]. The amount of UV-B radiation reaching the Earth’s surface has increased significantly due to the ozone depletion in the atmosphere [14]. UV-B radiation causes direct and indirect damage to cellular components, including DNA, RNA, proteins, and lipids, by generating reactive oxygen species (ROS) [15,16,17]; the latter can be scavenged by the enzymatic activity of superoxide dismutase, catalase, ascorbate peroxidase, and glutathione reductase, as well as non-enzymatically by ascorbic acid, alpha-tocopherol, carotenoids, and flavonoids [18]. Although plants are affected by UV radiation, low doses of radiation can be beneficial, by stimulating the synthesis of secondary metabolites with plant-protective properties, such as phenolics, carotenoids, anthocyanins, flavonoids, terpenes, and glucosinolates [19].

UV-B radiation has been shown to affect pollen functionality by reducing cell viability, germination, and pollen tube length [19,20,21,22]. However, the specific molecular targets of UV-B radiation on pollen are still unclear. In addition, the extent of damage caused by UV-B radiation appears to vary between different species. In one report, nineteen taxa of pollen grains were germinated in vitro and exposed to two levels of UV-B radiation; most of the species studied showed a reduced rate of pollen germination and tube growth [23]. In a study of 34 taxa, only five showed a negative effect on germination, while more than half had a significant reduction in pollen tube length, including *Nicotiana tabacum* L. [20]. *Brassica napus* L. pollen was found to exhibit a significant decrease in both germination and pollen tube length after UV-B exposure; the same study also showed that lipid peroxidation increased, and the antioxidant levels decreased [24]. Overproduction of nitric oxide (NO) in *Zea mays* L. pollen has been identified as a major contributor to pollen toxicity [25]. UV-B exposure increased the mutagenic activity and decreased the viability of developing *Vicia faba* L. pollen [26]. UV-B treatment in *Nicotiana plumbaginifolia* L. increased pollen tube germination, but only at low radiation doses [27]. The sensitivity of *Glycine max* L. pollen to UV-B was also found to be dose-dependent [28].

The goal of this study was to investigate the potential effects of UV-B radiation on *Nicotiana tabacum* L. pollen and to assess the extent to which these effects could be mitigated by expressing Dsup. The decision to analyze tobacco pollen was influenced by the vast knowledge available on this cellular system, as well as the availability of standardized transformation methods. To achieve this, the Dsup gene was cloned and inserted into a pollen-specific vector under the control of a pollen-specific promoter. Dsup expression and distribution were monitored using a downstream YFP gene. The Dsup-containing vector was introduced biolistically into tobacco pollen. UV-B stress was applied to both non-germinated pollen grains and pre-germinated pollen tubes. To assess UV-B-induced damage and the protective activity of Dsup, we measured the pollen tube length, the distance between the ‘male germ unit’ and the pollen tube tip, the distance between the tube apex and the nearest developed callose plug, the relative content of specific marker proteins, and the antioxidant activity. The results showed that expressing Dsup could mitigate some of the negative effects of UV-B exposure.

## 2. Materials and Methods

### 2.1. Collection, Storage and Use of Tobacco Pollen

The *Nicotiana tabacum* L. pollen used in this study was collected from plants grown annually in the Botanical Garden of Siena (via Mattioli 4, 53100 Siena, Italy). Seeds are available at the herbarium of the Botanical Garden. Following anther dissection in a controlled laboratory setting, the pollen was dehydrated with silica gel and stored at −20 °C. The pollen was then gradually acclimated to room temperature (25 ± 2 °C) before being placed in a humid chamber (70% RH) overnight to rehydrate. The pollen was germinated for the required time in a BK medium containing 12% sucrose [29].

### 2.2. Preparation of the Expression Plasmid

The plasmid vector pHD32 utilized for pollen transformation was provided by Prof. Benedikt Kost from Friedrich-Alexander-Universität in Erlangen-Nürnberg, Germany. The pHD32 plasmid is 4368 base pairs long and includes a potent pollen-specific promoter (LAT52), multiple restriction sites, the yellow fluorescent protein (YFP) gene, an origin of replication, and a selection marker that confers resistance to ampicillin. The Dsup gene (GenBank: LC050827.1; UniProt P0DOW4) was amplified from the pCXN2KS-Dsup construct (Addgene plasmid #90019; http://n2t.net/addgene:90019, accessed on 1 April 2024). This construct includes the coding sequence for the Dsup gene of *Ramazzottius varieornatus* [8]. Primers were used to introduce restriction sites for ApaI and NgoMIV at the 5′ and 3′ ends of the insert, respectively. The pHD32 plasmid and insert were digested with ApaI and NgoMIV at 37 °C for 4 h. The insert was purified directly using the GeneElute PCR Clean-up Kit (Sigma-Aldrich, Milano, Italy), while the plasmid was purified using the QIAquick Gel Extraction Kit (Qiagen, Milano, Italy) after running on a 2% agarose gel. Both purified products were quantified using Nanodrop (Thermo Scientific, Rodano, Italy). The recombinant vector resulting from this experiment was cloned into competent Escherichia coli cells, specifically the strain DH5alpha. The vector was then extracted using the GeneJET Plasmid Miniprep Kit from Thermo Scientific, quantified using Nanodrop, and stored at −20 °C.

### 2.3. Transient Pollen Transformation

The transformation of tobacco pollen with plasmid pHD32-Dsup was performed using the PDS-1000/He (Bio-Rad, Milano, Italia) system. The selected rupture disc was designed to withstand a pressure of 1100 psi [30]. The macrocarrier contained 1 µm diameter gold particles precoated with plasmid DNA (microcarrier). Each plate containing tobacco pollen was bombarded twice and rotated 90° between bombardments. 30 mg of gold particles were resuspended in 1 mL of cold 70% ethanol, vortexed for 3 min, and allowed to stand for 15 min at room temperature. After centrifugation at 16,000× *g* for 5 s, the supernatant was removed, and 1 mL of DEPC-H_2_O was added to the pellet. The latter was vortexed for 1 min and then allowed to stand for 1 min. After centrifugation at 16,000× *g* for 5 s, the pellet was washed twice with DEPC-H_2_O and resuspended in 500 µL of 50% sterile glycerol. Microprojectiles aliquots were sonicated for 2 min. Then, 10 µL of plasmid DNA (0.25 µg/µL), 25 µL of cold calcium chloride (2.5 M), and 15 µL of cold spermidine (0.1 M) were added. The mixture was vortexed for 30 s and allowed to stand on ice for 15 min. After centrifugation at 16,000× *g* for 5 s, the supernatant was discarded and 1 mL of cold 70% ethanol was added to the pellet. The solution was allowed to stand on ice for 15 min before further centrifugation. Then, 150 µL of chilled 100% ethanol was added to the pellet, which was vortexed for 30 s, and stored on ice until used. Each aliquot was sonicated and vortexed for a few seconds and 8 µL of the suspension was applied to the center of a macrocarrier. After inserting all components into the biolistic, the bombardment chamber was closed, and a vacuum of 27 mm Hg was created. The pressure was set to 1200 psi, which exceeded the strength of the ruptured discs. The transformation method was used without modification for all the analyses described below.

### 2.4. Pollen Germination and UV-B Stress

**Figure 1:**
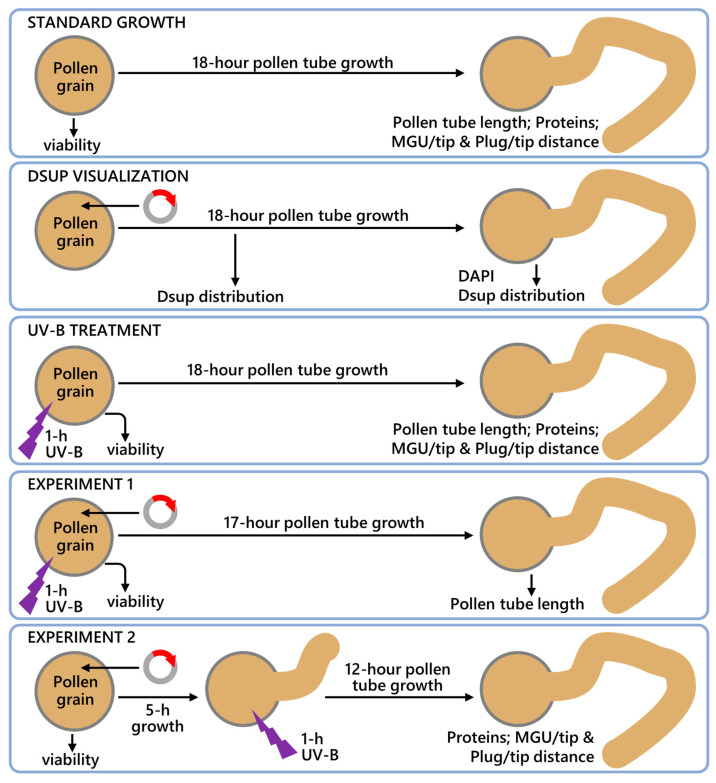
Summary of experimental procedures on tobacco pollen. Pollen under standard growth conditions was not subjected to UV-B stress or transformation; instead, it was germinated for 18 h. To visualize the Dsup distribution, pollen was transformed (the circle with the red arrow represents the Dsup-containing plasmid) and the Dsup-YFP signal was evaluated after 5 and 18 h, the latter in parallel with DAPI staining. The UV-B treatment consisted of exposing the pollen to UV-B (purple light) for 1 h; after 18 h of growth, the pollen tubes were evaluated for the parameters described in each subpanel. In Experiment 1, pollen grains were exposed to UV-B stress for 1 h immediately after transformation for Dsup expression and germinated for another 17 h. Experiment 2 involved transforming pollen grains to express Dsup, subjecting them to 1 h of UV-B stress after 5 h of germination, and then allowing them to germinate for another 12 h; at the end, several parameters of the pollen tubes were assessed.

Tobacco pollen was exposed to two different conditions. In Experiment 1, UV-B stress was applied to hydrated, non-germinated pollen grains for 1 h, and pollen viability was measured. Prior to UV-B treatment, a fraction of the hydrated pollen grains was transformed to express Dsup. The untreated control pollen, UV-B-stressed pollen, and Dsup-expressing pollen were all allowed to germinate for at least 18 h under standard conditions. In Experiment 2, a portion of the pollen grains was transformed and germinated alongside unprocessed control pollen in standard conditions. After five hours, both transformed and non-transformed pollen tubes were subjected to UV stress for an hour. They were then allowed to grow under standard conditions for a total of 18 h (Figure 1). UV-B stress was applied by exposing pollen (or pollen tubes) to TL20W/12 lamps (Philips, Milan, Italy), which emit at UV-B wavelengths and have been widely described in the literature [31]. Pollen was placed 15 cm under UV-B lamps in a climate chamber with a temperature of 23 °C. We found that longer distances between lamp and pollen had no consistent effect on pollen germination or tube growth, whereas longer application times (such as 2–3 h) had a more severe negative effect on pollen grains. These settings are based on both unpublished and published work from our lab [19,32]. Controls included the bombardment of pollen with DNA-free particles to test the effect on pollen viability, and with particles carrying plasmid DNA without the Dsup gene to test the specificity of the Dsup-YFP signal (Figure 1). Preliminary studies of bombardment with DNA-free particles showed no difference in response to UV radiation compared to untreated pollen, except for reduced viability. No other light sources were used, and pollen/pollen tubes were kept dark before and after UV-B exposure. The homogeneity of the UV-B radiation emitted by the lamps was verified daily using an 840 power meter with an 818-UV sensor (Newport Optical, Irvine, CA, USA). The UV-B biologically effective dose (BED), 25 KJ m^−2^ d^−1^, was calculated according to Correia et al. [33]. The UV-B treatment corresponds to a high dose of UV-B, which is naturally found in some regions of the earth’s surface [34]. The UV-B dose used in our assay is unlikely to be specific to tobacco pollen, but the male gametophyte of this species remains one of the best cell models for understanding the cytomolecular basis of the response.

### 2.5. Cell Viability Assay

The viability of pollen grains was assessed using the fluorochrome reaction (FCR) test with fluorescein diacetate (FDA) dye, following the protocol described by Heslop-Harrison et al. [35]. The FDA is taken up by living cells and converted by esterases into a fluorescent probe that labels viable cells. The BK-FDA solution was prepared by adding FDA drop by drop to the BK medium until the solution turned milky. Then, 20 µL of the BK-FDA solution was placed on a microscope slide, and 20 µL of pollen was added to each drop. Observations were made using a fluorescence microscope with a fluorescein filter (Zeiss Axiophot, Milano, Italy, 20× and 40× objectives). Images were captured with a Zeiss Axiocam MRm camera. The percentage of viable pollen was calculated by counting 200 grains per sample.

### 2.6. Analysis of Pollen Tube Length

Pollen tube length is a parameter used to evaluate the effect of abiotic stresses or potentially cytotoxic substances. Therefore, the length of 100 pollen tubes per sample was measured, and the analysis was performed in triplicate. Observations were made 18 h after the onset of germination using a Zeiss Axiophot light microscope (Milano, Italy) (20× and 40× objectives), and images were captured using a Zeiss Axiocam MRm camera. Pollen tube length measurements were performed using ImageJ software (v. 1.54i, released by the National Institutes of Health, MA, USA), calibrated against the reference scale bar.

### 2.7. Visualization of the Expression of Dsup

Pollen tubes expressing the Dsup gene were examined under a fluorescence light microscope (Zeiss Apotome) with a fluorescein filter (40× and 63× objectives). The expression of Dsup was indirectly monitored by examining the YFP signal. Pollen was examined at various stages of tube growth after at least 4–5 h of germination. At least 50 pollen tubes per sample were examined to obtain a consistent and reliable visualization. Analyses were performed in at least triplicate.

### 2.8. Analysis of the Position of Callose Plugs and Nuclei in the Pollen Tube

A 0.1% solution of aniline blue was used to visualize the position of callose plugs within the pollen tubes after 18 h of germination. Pollen was incubated with the probe for 5 to 10 min and observed under a fluorescence light microscope with a UV filter (Zeiss Axiophot, 20× and 40× objectives) to capture images with the Zeiss Axiocam MRm camera. Observations were made on 50 pollen grains per sample, and the distance between callose plugs was measured using ImageJ software. A minimum of three biological replicates were analyzed.

DAPI (4′,6-diamidino-2-phenylindole) was used to localize vegetative and sperm nuclei by binding to adenine-thymine-rich regions in DNA. The pollen was observed under a fluorescence light microscope with a UV filter (405 nm, Zeiss Axiophot, 20× and 40× objectives) after incubation with the probe for several minutes. At least 50 pollen tubes per sample (in triplicate) were considered for analysis. The distance between the nuclei and the tip of the pollen tube was measured using ImageJ software.

### 2.9. Evaluation of Polyphenols, Flavonoids, and Antioxidant Capacity

For each condition (CTRL, UV-B, +Dsup, UV-B + Dsup), 100 mg of pollen was weighed and germinated in a BK medium with 12% sucrose. After germination, the pollen was centrifuged at 13,000× *g* for 15 min. The supernatant was discarded, and the pollen from the pellet was transferred to a 2 mL tube. After adding 300 µL of 70% acetone, the pollen was lysed using a mixer mill MM 400 (Retsch GmbH, Pedrengo (BG), Italy, 30 Hz, 2 × 2 min). The lysate was centrifuged at 4000× *g* for 5 min, and the supernatant (extract) was transferred to another 2 mL tube. Three biological replicates were tested, and assays were performed in triplicate.

The ferric reducing antioxidant power (FRAP) assay was used to calculate the total antioxidant power [36]. To perform each reaction (in triplicate), 233 μL of 300 mM acetate buffer (pH 3.6), 23 µL of 10 mM TPTZ (2,4,6-tripyridy-s-triazine), 23 µL of 20 mM ferric chloride (FeCl_3_), and 2.5 µL of extract (with water as the blank) were combined. After that, the mix was incubated at 37 °C for 1 h, and the absorbance was read at 593 nm using a microplate reader (Thermo Fisher Scientific, Milano, Italy). The absorbance value was interpolated with the standard curve related to ferrous sulphate. Values were expressed in mmol of ferrous (Fe^2+^) equivalent per 100 g of pollen, presented as mean ± standard deviation.

The Folin–Ciocâlteu assay was used to determine the total polyphenols content (TPC) [37]. In each triplicate reaction, 25.5 µL of extract (distilled water for white), 203 µL of distilled water, 13 µL of F-C reagent (Sigma Chemical, St. Louis, MO, USA), and 38.5 µL of a sodium carbonate saturated solution (Na_2_CO_3_) were combined. The mix was incubated at 37 °C for 30 min. The absorbance values were measured at 795 nm using microplate readers (Thermo Fisher Scientific, Waltham, MA, USA). A calibration curve for gallic acid (Sigma Chemical, St. Louis, MO, USA) was used to calculate the TPC in mg of gallic acid equivalents (GAE) per 100 g of pollen, expressed as mean ± standard deviation.

Finally, the total flavonoids content (TFC) was determined using the aluminum chloride assay [38]. For each reaction (in triplicate), 28 µL of extract (water as the blank), 84 µL of 95% ethanol, 5.6 µL of 10% aluminum chloride, 5.6 µL of 9.8% potassium acetate, and 156 µL of distilled water were added. The mixture was then incubated at room temperature for 1 h, and the absorbance at 415 nm was measured using a microplate reader (Thermo Fisher Scientific, USA). The results were compared to a calibration curve generated using the quercetin standard (Sigma Chemical, St. Louis, MO, USA). The values were expressed as mg of quercetin equivalent (Qe) in 100 g of pollen.

### 2.10. Analysis of Marker Proteins by Immunoblotting

The pollen was allowed to germinate under constant agitation at 25 °C. After germination, it was collected by low-speed centrifugation (1000 rpm for 5 min). The resulting pellet was washed with an HEM buffer (50 mM Hepes pH 7.5, 2 mM EGTA, 2 mM MgCl_2_) containing 12% sucrose. The pollen in the pellet was then lysed with a Mixer Mill MM 400 (Retsch GmbH, 30 Hz, 2 × 2 min) using the HEM lysis buffer supplemented with 10 µg/mL protease inhibitors and 1 mM DTT. After lysis, the samples were centrifuged at 500× *g* for 10 min at 4 °C. The supernatant was then removed and centrifuged at 16,000× *g* for 60 min at 4 °C. The resulting supernatant was precipitated with 12% TCA in cold acetone for 1 h at −20 °C. The precipitated proteins were then centrifuged at 13,000× *g* for 10 min at 4 °C. The resulting pellet was washed with cold acetone and centrifuged at 13,000× *g* for 10 min at 4 °C. After centrifugation, the pellet was allowed to dry for 10 min. Then, 20 µL of 0.2 M NaOH was added to neutralize the TCA. Finally, the pellet from each sample was resuspended in 150 µL of Laemmli sample buffer 1× and stored at −20 °C. The proteins were separated by 1D electrophoresis on 12% polyacrylamide gels using the TGX Stain-Free Kit from Bio-Rad (Milano, Italy). The samples were separated at 200 V using a TGS running buffer. The proteins were then made fluorescent using the ChemiDoc MP Imaging System (Bio-Rad) and transferred to Bio-Rad nitrocellulose membranes using the Trans-Blot Turbo (Bio-Rad) for 7 min. The transfer was verified by observing the membrane on the ChemiDoc MP Imaging System. Subsequently, the nitrocellulose membranes were blocked with a 5% membrane-blocking solution (Cytiva, Buccinasco, MI, Italy) for 1 h. After blocking, the membranes were washed with TBS and incubated for 1 h with primary antibodies: antibody to 75-kD HSP70 (AS081371, Agrisera, Vännäs, Sweden) diluted 1:5000; antibody to 43-kD actin (Sigma, Milano, Italy, 10-B3) diluted 1:3000; and antibody to 50-kD tubulin (Sigma, B-5-1-2) diluted 1:5000. The membranes were washed twice for 5 min in TBS. The secondary antibodies were then added. These included the StarBright Blue 700 goat anti-rabbit IgG antibody (Bio-Rad) diluted to 1:2500 and the StarBright Blue 520 goat anti-mouse conjugated antibody (Bio-Rad) diluted to 1:2500. After incubation, the membranes were washed twice for 5 min with TBS. Finally, the signals from both StarBright Blue 700 and StarBright Blue 520 were analyzed using the ChemiDoc MP Imaging System (Bio-Rad, Milano, Italy) with automatic exposure selected in both cases.

### 2.11. Western Blot Analysis Using Image Lab Software

Immunoblotting results were quantified using Bio-Rad’s Image Lab software (v. 6.1) to more accurately assess the levels of selected immunoreactive proteins. Membrane images obtained from Stain-Free protein staining were normalized to a reference lane selected by the operator. The immunoblotting signals were then normalized to the protein content of each lane. This eliminates the problem of different protein loading in individual electrophoretic blots.

### 2.12. Statistical Analysis of Data

The experimental data were analyzed using GraphPad Prism v.10 software (https://www.graphpad.com/; Boston, MA, USA). The t-test was used for two sets of data, while one-way ANOVA, followed by Šidák’s multiple comparison test, was used for more than two groups. The data are significant for * *p* < 0.05, ** *p* < 0.009, *** *p* < 0.0006, **** *p* < 0.0001.

## 3. Results and Discussion

### 3.1. Pollen Viability Assessed by FDA Test

**Figure 2:**
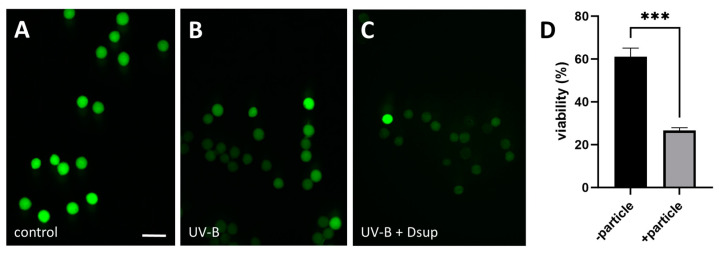
FDA test results for tobacco pollen grains. (**A**) Control pollen; (**B**) UV-B-treated pollen; (**C**) UV-B-treated and bombarded pollen. The bar in panel A (50 µm) refers to all three images. Viable pollen grains are bright green in color, whereas non-viable pollen grains are barely visible. (**D**) Calculation of pollen viability before and after particle bombardment. The difference is significant (*** *p* < 0.001).

In the first step, we measured pollen viability using FDA on three samples: control, UV-B-treated, and transformed/UV-B-treated pollen (as in ‘Experiment 1’). In this case, the transformation consisted of bombarding the pollen with microcarriers that did not carry the plasmid. This analysis revealed that pollen had a viability rate of 82%, which was acceptable, considering that the pollen was frozen and not fresh. We found that the pollen exposed to UV-B had a lower viability rate (63%), while the pollen exposed to UV-B and bombarded with biolistic had the lowest viability rate, approximately 35%. These results were obtained by analyzing multiple images, such as those shown in Figure 2A–C, which demonstrate the effects of UV-B stress and biolistic transformation on pollen viability. The data clearly showed a decrease in the number of bright pollen grains following UV-B stress and bombardment. This result is not surprising because stressful conditions are well-known to affect pollen viability [39]. As an additional test, we measured the change in pollen viability before and after particle bombardment on the same sample. The findings show that biolistic transformation significantly reduced pollen viability from 60% to around 30% (Figure 2D).

### 3.2. Localization of Dsup in the Pollen Tube

**Figure 3:**
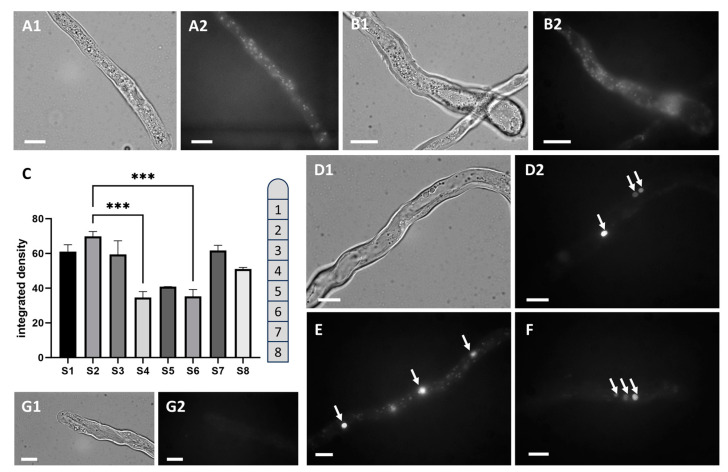
Localization of Dsup in tobacco pollen tubes observed by both light and fluorescence microscopy. (**A1**,**A2**,**B1**,**B2**) Dsup was detected as multiple fluorescent spots scattered along the pollen tubes after 3–4 h of growth. (**C**) Measurement of fluorescence intensity in 10-µm segments (S1–S8) from the tube tip (as represented by the segmented pollen tube on the right). Values are significant (*** *p* < 0.001). (**D1**,**D2**) Light and fluorescence microscopy images showing consistent aggregates of Dsup-YFP (arrows) after 6–7 h of growth. (**E**,**F**) Fluorescence microscopy showing distinct fluorescent spots (arrows), sometimes very close together. (**G1**,**G2**) Control pollen tube in light and fluorescence microscopy with very faint background. Bars: 10 µm.

The subcellular localization of Dsup was determined by analyzing the distribution of the fused YFP. This specific analysis was performed on pollen transformed for Dsup expression but not exposed to UV-B stress. Consequently, these experimental data were also used to assess any damage caused by the transformation process alone. The length of the pollen tube, i.e., its growth capacity, was not significantly altered, but only the viability of the pollen grains, as already shown in Figure 2D. Microscopy images revealed that protein distribution varied according to the time of expression. Figure 3(A1,A2), which depict the same pollen tube under both visible and fluorescent light, show the distribution of Dsup 3–4 h after germination. Dsup appeared as a uniform distribution of bright dots throughout the pollen tube. Figure 3(B1,B2) depict another pollen tube with a diffuse distribution of Dsup, which resembles small protein clusters randomly distributed throughout the tube. ImageJ was used to measure fluorescence intensity in 10-µm segments along pollen tubes (Figure 3C). The first segments closest to the apex had a stronger signal, while the intensity decreased further away from the apex. Figure 3D–F depict the typical distribution of Dsup in pollen tubes that have been germinated for a longer period of time (approximately 6–7 h). In this case, prominent fluorescent bodies were seen alongside diffuse cytoplasmic fluorescence, occasionally in clusters of two or three (arrows); they were distributed throughout the pollen tube but frequently clustered together (as shown in Figure 3(D2,F)). Figure 3(G1,G2) show control pollen tubes bombarded with gold particles that do not carry the plasmid, resulting in a faint and diffuse fluorescent background. 

**Figure 4:**
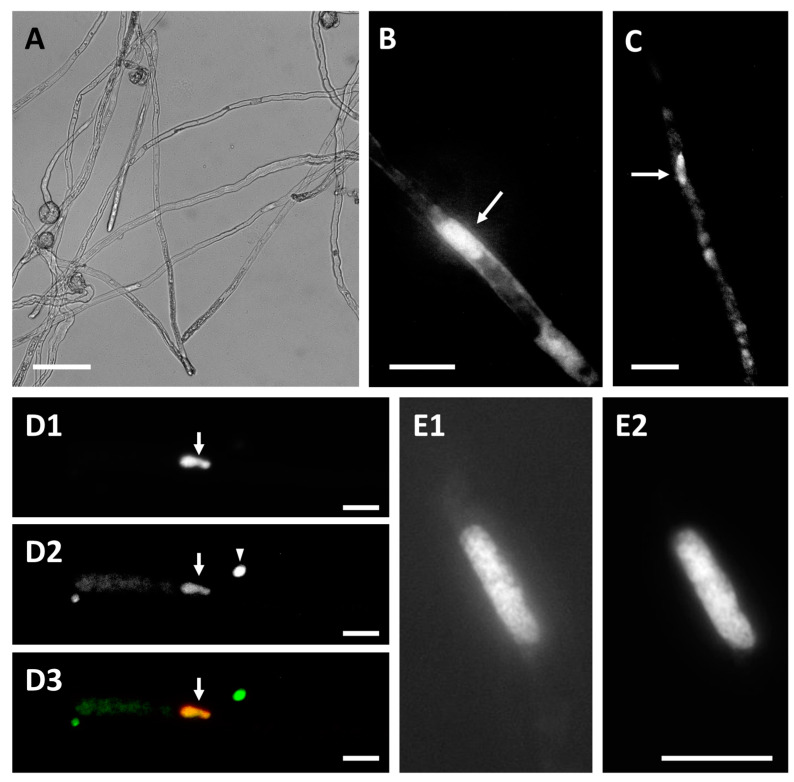
Double colocalization of Dsup and nuclei in pollen tubes grown for 18 h. (**A**) Light microscopy image of transformed tobacco pollen tubes grown overnight. Bar: 100 µm. (**B**,**C**) Two pollen tubes labeled with Dsup-YFP; the arrows indicate consistent accumulation of Dsup in nucleus-like structures. Bars: 20 µm. (**D1**) A pollen tube stained with DAPI; (**D2**) the corresponding pollen tube in the Dsup-YFP channel; (**D3**) merging of the previous images showing the colocalization of Dsup with the DAPI-stained nucleus. The arrow indicates overlapping DAPI/Dsup-staining of the nucleus; the arrowhead indicates a Dsup-labeled structure that is not stained by DAPI. (**E1**) Detail of a Dsup-labeled nucleus; (**E2**) DAPI-stained pollen tube from (**E1**). Bars: 20 µm.

We also examined and compared the fluorescent signal of Dsup to that of DAPI after overnight pollen tube growth to determine the relationship between Dsup distribution and the location of vegetative and sperm nuclei. Figure 4 shows that Dsup was consistently accumulated in large fluorescent bodies (Figure 4B,C, arrows), in addition to the diffuse signal in the pollen tube cytoplasm. To confirm the correlation with nuclei, samples were counterstained with DAPI (Figure 4D), revealing that the Dsup signal (D2) overlapped with the DAPI-stained vegetative nucleus (D1). The merging of D1 and D2 in Figure 4(D3) confirmed colocalization (arrow). The arrowhead indicates a Dsup-stained body that has not been stained with DAPI. A high magnification of the nucleus revealed that DAPI staining and Dsup labeling colocalized in the pollen tube nuclei, albeit in slightly different patterns (Figure 4(E1,E2)). The distribution of Dsup in the nuclear material is consistent with previous reports in the literature about neurons, implying that the ultimate target of the protein is the nucleus, where it will exert its protective effect [40]. In our case study, Dsup localization in the pollen nucleus occurred only after overnight or several hours of tube growth, implying that Dsup expression and accumulation must be consistent for the protein to be targeted to the nucleus.

### 3.3. UV-B-Stressed and Transformed Pollen Grains Produce Pollen Tubes That Grow Consistently

**Figure 5:**
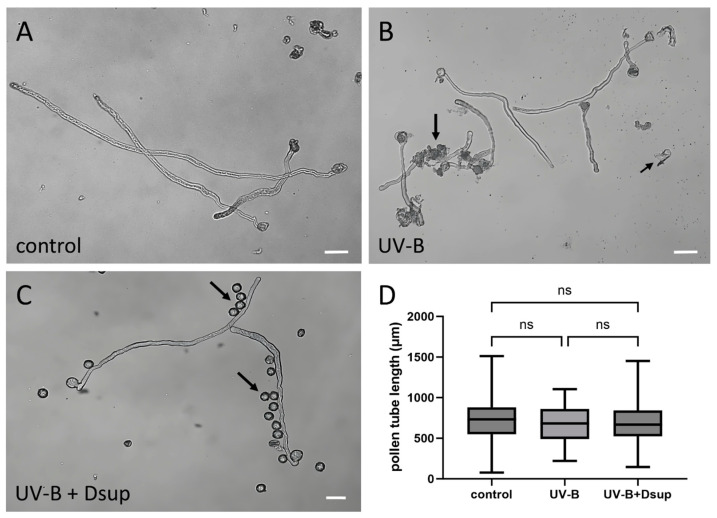
Analysis of tobacco pollen tube length after transformation with Dsup and treatment with UV-B. The pollen germinated for 18 h. (**A**) The control group of pollen germinated under normal conditions, without UV-B treatment or transformation. (**B**) Pollen treated with UV-B and then germinated. Note that some pollen grains were damaged (arrow). (**C**) Pollen transformed with Dsup and treated with UV-B before germination; some pollen grains did not germinate (arrows). (**D**) Graph depicting pollen tube growth measurements in three conditions: control, UV-B treatment, and Dsup transformation, followed by UV-B treatment. The growth rate of pollen tubes was similar in all three cases, and no statistically significant differences in tube lengths were detected (ns). Bars: 50 μm.

Figure 5 depicts the effects of UV-B treatment on pollen tube length before and after transformation with Dsup, as described in Experiment 1. The control group consisted of pollen grains that germinated without any treatment, neither receiving UV-B irradiation nor undergoing transformation with Dsup (Figure 5A). After 18 h of germination, the pollen tube length in the control samples was approximately 800 µm, as expected for tobacco pollen. In the group of pollen grains exposed to UV-B radiation prior to germination (Figure 5B), some damaged pollen grains were observed (arrows), indicating that exposure to UV-B radiation has a negative effect on pollen viability (as shown in Figure 2). However, there were no statistical differences in pollen tube length between the UV-B-stressed pollen and the control groups. In the sample containing pollen grains transformed with Dsup and exposed to UV-B radiation, some ungerminated and possibly dead pollen grains were again visible (Figure 5C, arrows). Even in this case, the pollen tubes were the same length as the control samples and the pollen group exposed only to UV-B. Figure 5D depicts the pollen tube growth rates in all three experimental cases. Although UV-B stress and transformation resulted in a fraction of ungerminated pollen grains, those pollen grains that did germinate produced pollen tubes with statistically comparable growth and length as the control samples.

According to published studies, UV-B stress has little effect on pollen tube length when applied to pollen grains. In the case of hazelnut pollen exposed to UV-B at different distances and times, the application of 1 h UV-B stress produced minor effects on pollen tube length [19]. Even in canola, UV-B had the most consistent effects on pollen tube length when applied for more than an hour before germination [41]. Data collected from maize pollen confirmed that the most consistent effects on pollen tube length were found after 40–90 min of UV-B exposure [42]. In a study of 34 taxa, *Nicotiana tabacum* pollen was exposed to low and high UV-B radiation for 180 min, and its germination capacity was reduced by only 5–10% compared to the control [20]. As a result, all data show that exposing ungerminated pollen grains to UV-B for a limited time reduces pollen grain viability and germination capacity while having little or no effect on pollen tube growth. This implies that UV-B stress may influence the mechanism of pollen grain germination. However, pollen grains that recovered efficiently from UV-B stress showed no defects in pollen tube growth rate, implying that UV-B stress has no effect on pollen tube growth when applied prior to germination. Similarly, the transformation for Dsup expression prior to pollen germination and UV-B treatment had no effect on pollen tube growth, either positively or negatively. We hypothesize that Dsup cannot recover the reduced viability and germination capacity induced by UV-B to ungerminated pollen grains because the gene is still under-expressed. This hypothesis is also supported by previously discussed evidence that Dsup accumulates in the nucleus of the pollen only after many hours of germination, implying that the protein has been expressed consistently.

### 3.4. Dsup Transformation and Expression Reduce the Negative Effects of UV-B Stress on Developing Pollen Tubes

**Figure 6:**
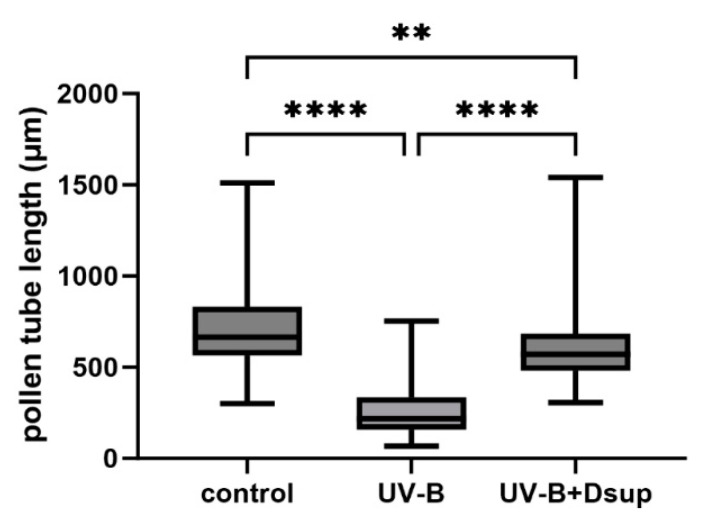
Measurement of pollen tube length under the conditions described in Experiment 2. Pollen grains were transformed and germinated for 4–5 h prior to UV-B exposure. The graph depicts the growth rate of pollen tubes in three cases (control, UV-B stress only, and Dsup transformation followed by UV-B). UV-B treatment significantly reduces pollen tube elongation (**** *p* < 0.0001). UV-B treatment with Dsup transformation partially restores pollen tube growth compared to the control (** *p* < 0.01), sometimes even exceeding it.

Figure 6 depicts the pollen tube length measurements from Experiment 2, which involved exposing pollen to UV-B radiation after 4–5 h of germination. The sample was divided into three groups: a control group that received no treatment, a group that only received UV-B radiation, and a third group in which the pollen grains were transformed to express the Dsup gene, and the resulting pollen tubes were exposed to UV-B stress 4–5 h after germination. The results show that UV-B radiation significantly reduced the length of pollen tubes when compared to both the control sample and the pollen sample that had undergone UV-B stress and transformation. The results confirm that UV-B radiation affects tobacco pollen, specifically the length of the germinating pollen tube. This result differs from Figure 5 and may indicate that pollen grains are more protected against UV-B stress due to protective cell walls that are more resistant to external agents than the pollen tube cell wall. Other studies have shown that pollen tubes exposed to UV-B radiation had a significant reduction in growth rate [20,21,22], with the effect becoming more pronounced as the duration of UV-B exposure increased. As a result, these findings support the hypothesis that the pollen tube is more susceptible to UV-B stress than the pollen grain. Our results also show that when pollen grains were transformed to express Dsup and allowed to germinate for 4–5 h before being subjected to UV-B stress (Experiment 2), the length of pollen tubes measured at the end of germination was comparable to, if not longer than, that of the control pollen. This suggests that the expression of the Dsup gene partially compensated for the inhibitory effects of UV-B stress on pollen tube growth, and that the ameliorative capacity of Dsup occurs when the Dsup gene is expressed for a few hours prior to UV-B stress, allowing Dsup to accumulate in sufficient levels to protect cells.

### 3.5. Dsup Expression Minimizes the Negative Impact of UV-B on the Distance between the Newly Formed Callose Plug and the Pollen Tube Tip

**Figure 7:**
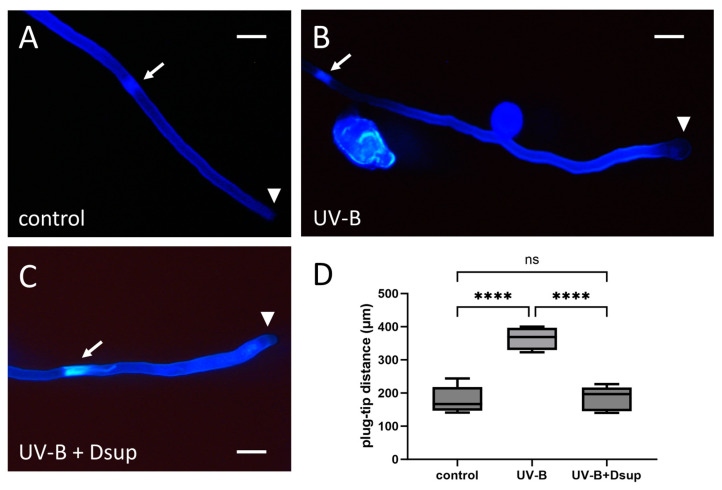
Effect of UV-B treatment on the position of the last formed callose plug in tobacco pollen tubes with/without Dsup expression (Experiment 2). (**A**) Control pollen tubes stained with aniline blue. The arrow indicates the last callose plug formed, while the arrowhead indicates the tip of the pollen tube. (**B**) Pollen tubes treated with UV-B; again, the arrow indicates the final callose plug and the arrowhead indicates the pollen tube tip. (**C**) Pollen tubes expressing Dsup, then treated with UV-B; the arrow indicates the final callose plug and the arrowhead indicates the tube tip. (**D**) Graph depicting the position of the last callose plug in the three cases studied. Control samples have uniform distances (around 200 μm on average). UV-B stress resulted in a value of 350–400 μm. After transformation, the data were homogeneous and comparable to the control. The control sample differed statistically from the UV-B treatment (**** *p* < 0.0001), as did the UV-B-treated sample, which differed from the UV-B-transformed sample (**** *p* < 0.0001). The control did not differ statistically from the treated or transformed samples (ns).

Figure 7 depicts the distance between the pollen tube tip and the last fully formed callose plug. This distance, which is critical for proper tube development, was measured in pollen tubes under various experimental conditions, including control pollen (A), UV-B-treated pollen tubes (B), and UV-B-treated pollen tubes germinated from Dsup-transformed pollen grains (C) (Experiment 2). The control sample demonstrated consistent results, with pollen tubes depositing the final callose plug at an average distance of 200 µm from the apex, as expected. The pollen sample transformed with Dsup and treated with UV-B did not differ significantly from the control, with an average distance of around 200 µm from the apex. In contrast, the UV-B-treated sample differed significantly from the control and Dsup-transformed UV-B-stressed pollen. The distance between the last callose plug and the apex was around 350–400 µm (Figure 7D).

The ameliorative effect of Dsup expression is significant because tube growth is dependent on the regular deposition of callose plugs and the regular distance between the last formed callose plug and the tube tip. In fact, callose plugs are regularly deposited in growing pollen tubes to separate the active cytoplasm from the vacuolar region; their primary function is to reduce total cell volume, thus regulating the turgor pressure required for the pollen tube’s apical and invasive growth mechanism [43]. The mechanisms governing the periodicity of callose plug deposition are not yet understood. The callose plugs are believed to be produced by local activation of callose synthase (CalS) in the pollen tube plasma membrane [44,45]. CalS is thought to be inserted into the plasma membrane in an inactive form and then converted to the active form through proteolytic processes [46]. The cytoskeleton, particularly the microtubules, is likely to play an important role in CalS accumulation in the distal regions of pollen tubes, which is necessary for callose plug formation. Aidemark et al. [47] found that oryzalin, a microtubule depolymerizing chemical, inhibited CalS activity in Arabidopsis and tobacco cells, implying that intact microtubules are required for quantitative callose synthesis. Furthermore, although callose plug synthesis can occur in the absence of microtubules, the number of plugs synthesized is reduced after microtubule depolymerization, which also affects the regularity of deposition [48]. Native 2D electrophoresis, CalS complex isolation, and far Western blot analysis all confirmed the interaction of CalS with tubulin and cortical microtubules [4]. Furthermore, electron microscopy immunolabeling revealed that CalS and microtubules colocalized via microtubule-associated vesicles containing CalS. The data from previous studies show that the regular deposition of callose plugs is dependent on the consistent accumulation/activation of CalS, which is dependent on proper microtubule organization. Because the distribution of microtubules is sensitive to UV-B radiation [49], UV-B stress may disrupt the regular deposition of callose plugs by indirectly influencing microtubule organization. More research is needed to understand how UV-B stress influences this periodicity and the underlying molecular mechanism. Because UV-B treatment alters tubulin levels, while Dsup expression restores proper tubulin content (see below), we propose that tubulin dynamics and levels influence callose plug deposition, and that UV-B stress influences callose plug deposition by altering tubulin levels, with CalS serving as a connecting ring between tubulin/microtubules and callose plugs. We also propose that Dsup expression may mitigate both effects.

### 3.6. Dsup Expression Does Not Reverse the Effects of UV-B on the Distance between the “Male Germ Unit” and the Apex of the Pollen Tube

**Figure 8:**
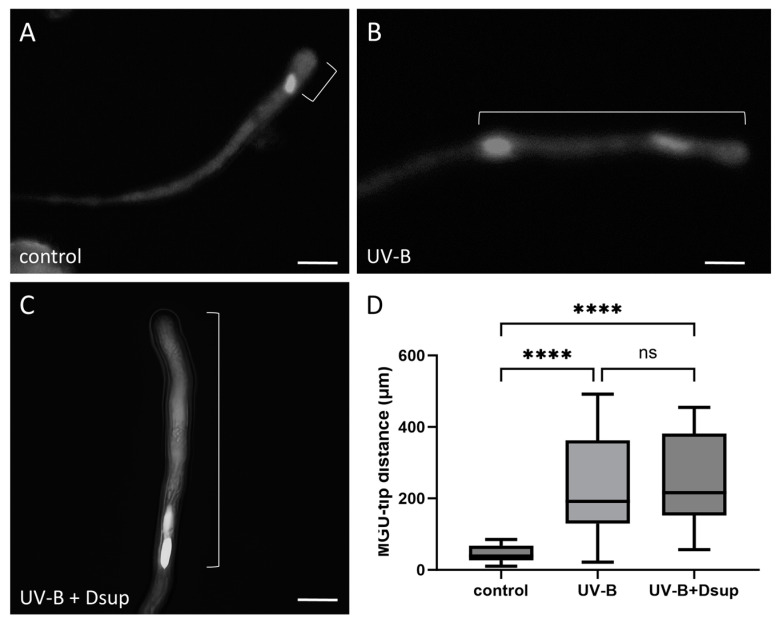
Analysis of the effect of UV-B stress on the position of the male germ unit in tobacco pollen tubes, both with and without transformation with Dsup (Experiment 2). (**A**) Control pollen tubes stained with DAPI. (**B**) UV-B-treated pollen tubes. (**C**) Dsup-transformed pollen tubes subjected to UV-B treatment. The square bracket indicates the distance between the MGU and the tip of the tube. (**D**) Measurements of the position of the MGU relative to the tube tip in the three cases studied. In control samples, the “MGU/tip” distance is uniform and averages around 40 μm. UV-B-stressed samples exhibited data ranging from 200 to 400 μm. After transformation, the data remained highly heterogeneous, with an average of 250 μm. The control differs significantly from the UV-B-treated and transformed samples (**** *p* < 0.0001). There was no difference between the UV-B-treated and transformed samples (ns).

Next, we determined the distance between the pollen tube tip and the male germ unit (MGU) (Figure 8) under the conditions of Experiment 2. The hypothesis was that UV-B radiation would affect MGU transport rates. The experiment was carried out under three conditions: untreated control pollen (Figure 8A), UV-B-irradiated pollen tubes (Figure 8B), and UV-B-irradiated pollen tubes germinated from pollen grains that had previously been transformed with Dsup (Figure 8C). Figure 8D shows significant differences in the MGU/tip distance between the UV-B-treated and UV-B/Dsup-transformed samples compared to the control (200–400 µm vs. 40 µm). Furthermore, the control samples were highly homogeneous, with a short distance between MGU and apex (average 40 µm). This finding reinforces the need for precise synchronization of MGU transport and pollen tube elongation. When this result is compared to the distance measured between the apex and the last formed callose plug (as shown in Figure 7D), it is clear that the last developing callose plug is deposited much further from the tube apex than the distance between the apex and the MGU. However, this ratio of distances is not always maintained in UV-B-treated samples because the distance between the “last formed callose plug/apex” and the distance between “MGU/apex” are quite close. If the MGU is excessively near to the last developing callose plug, sperm cells may become trapped and separated from the living tube segment, causing pollen tubes to lose their primary function.

The movement of the vegetative nucleus and sperm cells (the MGU) to the apex during pollen tube development is critical for successful fertilization; any delay in this process can result in the failure of fertilization. The two cytoskeletal systems of pollen tubes (actin filaments and microtubules) include motor proteins that transport cargo via ATP hydrolysis [50]. Myosins, which are actin-associated motor proteins, are primarily involved in organelle movement within the pollen tube, possibly including the MGU [51]. The role of microtubules and the motor protein kinesins in MGU movement is less clear. However, it has been shown that without microtubules, MGU progression is severely slowed, and the actin filaments also adopt an abnormal organization, presumably making migration even more difficult. Thus, cooperation between both cytoskeletal components is required [5,48]. Recent research has also shown that LINC complexes (linkers of the nucleoskeleton and cytoskeleton) play an important role in nuclear migration because they connect the nucleus to the cytoskeleton and/or motor proteins [52]. The LINC complex is composed of SUN proteins (associated with the inner nuclear membrane) and KASH proteins (associated with the outer nuclear membrane). These proteins interact both directly and indirectly with cytoskeletal elements. The ‘WIT-WIP-SUN LINC complex’ in pollen is of particular interest because mutants in WIT or WIP show a significantly greater distance between the MGU and the apex than the wild type, indicating a critical role of this complex in MGU migration [53]. Calcium and ROS levels in Arabidopsis have been shown to influence the action of these proteins, as well as the distance between the MGU and the apex [54]. Based on these findings, the increased distance between the MGU and the apex in pollen tubes developed from UV-B-stressed pollen grains could be attributed to changes in components such as the cytoskeleton and calcium ions, as well as ROS levels. The latter have been shown to be affected when the pollen tube is subjected to abiotic stress [55,56]. Our results also show that Dsup expression is unable to repair the damage caused by UV-B stress to the “MGU/tip” distance, implying that while the ameliorative capacity of Dsup can recover the correct positioning of callose plugs, it cannot correct UV-B-induced defects in MGU positioning.

### 3.7. Dsup Expression Has Consistent Effects on Specific Marker Proteins

**Figure 9:**
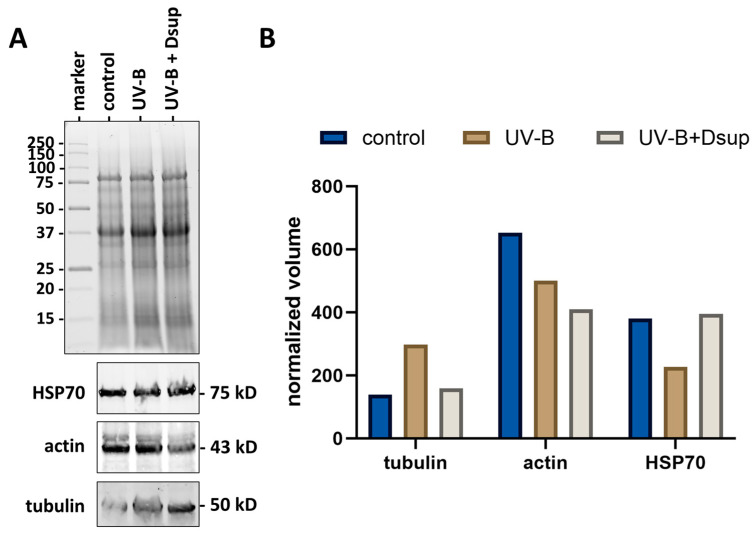
Immunoblotting analysis of protein extracts from control and UV-B-treated pollen with or without Dsup transformation. (**A**) Gel electrophoresis of the three samples analyzed. Marker: molecular weight standard in kD. Immunoblots with anti-HSP70 antibody, anti-actin antibody, and anti-tubulin antibody are shown below the gel. (**B**) Quantitative analysis using Image Lab software. The intensity of the immunoreactive bands was normalized to the total protein in each lane.

To better understand the response of tobacco pollen to both UV-B stress and Dsup transformation, we measured the levels of some specific proteins required for pollen tube growth. HSP70, a typical anti-stress protein, was previously described in heat-stressed pollen tubes [57,58]. Tubulin and actin are basic cytoskeletal proteins [59]. Figure 9 depicts the results and quantification of immunoblots against tubulin, actin, and HSP70 proteins in pollen tubes under experimental conditions 2. Figure 9A illustrates the electrophoretic analysis of three samples: control pollen, pollen stressed with UV-B radiation, and pollen treated with UV-B and transformed for Dsup expression. The protein profile appears to be consistent across all three samples. The immunoblotting results for the three proteins tested are shown at the bottom of the gel: HSP70 (75 kD in pollen), actin (43 kD), and tubulin (50 kD). Although the immunoblotting data already indicated changes in the three samples analyzed, we used relative quantification to obtain a more accurate assessment by normalizing the three samples and, as a result, each immunoblot. Figure 9B depicts typical quantification results, demonstrating that tubulin levels increased in the pollen exposed to UV-B stress. The tubulin levels in pollen treated with UV-B and expressing Dsup were comparable to those in the control group. Actin levels varied between the control and UV-B-stressed groups, as well as between the control and the Dsup-transformed UV-B-stressed group, and they decreased consistently. In contrast, the HSP70 content decreased after UV-B stress but returned to control levels when UV-B-stressed pollen expressed Dsup.

As previously stated, changes in tubulin levels may be linked to a different pattern of callose plug deposition [4,45,48]. Furthermore, microtubules in the pollen tube play a role in both the transport of the male germ unit and the local slow movement of organelles and vesicles [60,61]. The two activities (sperm transport and organelle/vesicle movement) are not necessarily linked, because pollen tubes can grow even in the absence of sperm cells [62]. We found that UV-B stress changed the position of the male germ unit, which was not restored by Dsup expression; however, Dsup expression did partially restore the pollen tube growth rate. This allows us to hypothesize that recovering tubulin levels through Dsup expression can restore pollen tube growth but not male germ unit movement, providing additional evidence that sperm cell movement is unrelated to pollen tube growth. It is important to note that Dsup expression did not restore actin levels, which could explain why the pollen tube growth rate did not recover completely.

The level of Heat Shock Protein 70 (HSP70), a protein with refolding properties, is known to be higher during the early stages of pollen development, but decreases as the maturation process progresses, suggesting a possible role for HSP70 in the early stages of pollen development [57,63]. The importance of HSP70 in early pollen development is most likely due to the distinct functions and structures of the pollen grain and pollen tube. The pollen grain is a cellular structure that can withstand harsh environmental conditions, most likely due to its thick and specialized cell wall. In contrast, the pollen tube has a thinner cell wall and a shorter lifespan, making it more vulnerable to abiotic stress, such as UV stress [64]. In experiments in which pollen tubes were exposed to heat stress, the basal level of HSP70 did not change as the temperature increased, indicating that a basal level of HSP70 expression is typically required and sufficient to protect the pollen grain and pollen tube [65]. Similarly, in our case study, the HSP70 levels did not increase and may even decrease, indicating that UV-B stress prevents HSP70 accumulation. Despite being exposed to UV-B stress, the pollen expressing Dsup had HSP70 levels similar to the control. Thus, it is possible to hypothesize that Dsup regulates HSP70 expression to provide indirect pollen protection.

### 3.8. Expression of Dsup Significantly Increased Antioxidant Capacity

**Figure 10:**
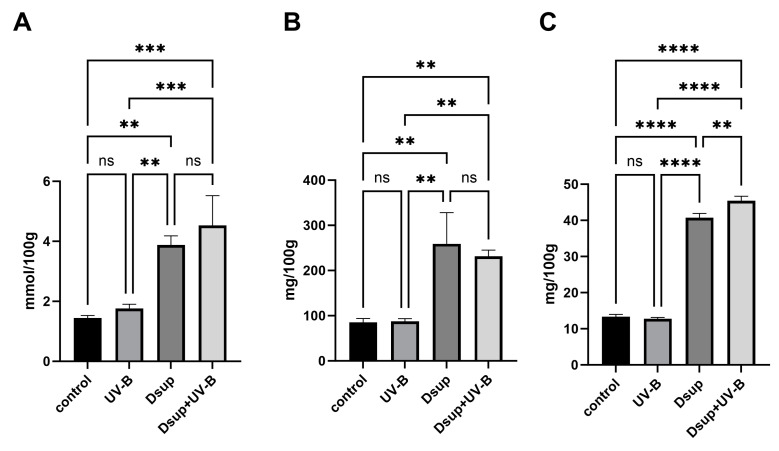
Antioxidant, polyphenol, and flavonoid content of control pollen tubes, UV-B-stressed pollen tubes, and Dsup-expressing pollen tubes. (**A**) FRAP assay for antioxidant content; (**B**) Folin-Ciocalteau test for polyphenol content; and (**C**) aluminum chloride test for flavonoid content. Control: untreated pollen tubes. UV-B: UV-B-stressed pollen tubes after 5 h of growth. Dsup: pollen tubes that express the Dsup gene but have not been exposed to UV-B radiation. Dsup + UV-B: Dsup-expressing pollen tubes exposed to UV-B after 5 h of growth (** *p* < 0.01; *** *p* < 0.001; **** *p* < 0.0001; ns: not significant).

To better understand how Dsup might help pollen recover from UV-B stress, we investigated the antioxidant, polyphenol, and flavonoid content of pollen tubes from three different studies (Figure 10). The FRAP method demonstrated that pollen tubes exposed to UV-B stress (Experiment 2) had antioxidant levels comparable to control samples (Figure 10A). Interestingly, the pollen tube sample that was not exposed to UV-B but expressed Dsup had significantly higher antioxidant content than the control and UV-B-stressed samples. The identical results were obtained in UV-B-stressed pollen tubes after inducing Dsup expression (Dsup + UV-B). A nearly identical trend was observed for polyphenol content determined using the Folin-Ciocalteau method. The polyphenol levels in the control sample and UV-B-stressed pollen tubes were comparable; however, Dsup-expressing pollen tubes showed significantly higher levels than the control (Figure 10B). The flavonoid content determined by the aluminum chloride assay followed a similar pattern as that of antioxidants and polyphenols (Figure 10C). Overall, the three tests revealed that the UV-B-stressed pollen tubes did not exhibit significantly higher levels of polyphenols, antioxidants, or flavonoids than the control after 18 h of growth. Nonetheless, expressing Dsup in both UV-B-stressed and unstressed pollen tubes resulted in a significant increase in antioxidants, polyphenols, and flavonoids, implying that Dsup expression improves pollen tube antioxidant defenses.

Pollen has a high antioxidant content, which is why it is frequently recommended as an essential component of the diet [66,67]. The high content of flavonoids (flavonols and anthocyanins) promotes pollen tube growth by lowering ROS levels and mitigating their adverse effects [56]. A few studies in the literature describe how UV-B radiation affects the antioxidant capacity of pollen, and they all conclude that UV-B radiation either has no effect or reduces it. It is important to note that pollen from different species can have varying levels of antioxidant activity, even when exposed to high UV radiation, such as those found in desert areas [68]. For example, maize pollen exposed to UV-B radiation showed an increase in reactive oxygen species and lipid peroxidation, resulting in a decrease in antioxidant capacity [42]. Additionally, a study on hazelnut pollen exposed to UV-B radiation found that the total polyphenol and flavonoid content decreased after three hours of treatment [19]. When Dsup is expressed in cells, it is expected to boost their antioxidant response, owing to its role in DNA protection, as reactive oxygen species have been shown to damage DNA. This was demonstrated by expressing Dsup in Drosophila [69] and animal cells [10] and assessing their resistance to UV-B radiation and oxidative stress. We hypothesize that increasing the antioxidant barrier, as demonstrated by Dsup expression, may provide a biochemical foundation for restoring both growth rate and callose plug deposition in pollen tubes under UV-B stress.

## 4. Conclusions

The data presented in this study demonstrate that the transformation of tobacco pollen grains to express the tardigrade Dsup gene can improve pollen tube tolerance to UV-B radiation. The positive effects highlighted are limited to specific aspects of pollen tube growth. First, to achieve these protective effects, the pollen tube must be properly developed, resulting in adequate Dsup expression. This restores pollen tube growth rate and callose plug spacing. The two aspects are related because pollen tube growth depends on both active vesicle secretion at the tip and proper turgor pressure, which is most likely associated with correct callose plug deposition. The protective action of Dsup is reflected in an increase in the content of antioxidants, polyphenols, and flavonoids, all of which have protective properties, especially antioxidant effects. This increase may protect the pollen grain and pollen tube from the reactive oxygen species produced by UV-B radiation. Although the tardigrade Dsup gene is heterologous to tobacco pollen, it protects against UV-B stress. Other processes involved in pollen tube growth, such as calcium ion and proton levels, cytoskeletal system organization, and deposition of other cell wall components, could be measured to determine the additional protective effect of Dsup expression.

## 5. Patents

A part of the manuscript content is object of Italian Patent Application No. 102024000010723 filed on 13 May 2024.

## Data Availability

The data presented in this study are available upon request from the corresponding author, as part of the work is under patent review.
